# Assessing the Impact of Green Transformation on Ecological Well-Being Performance: A Case Study of 78 Cities in Western China

**DOI:** 10.3390/ijerph191811200

**Published:** 2022-09-06

**Authors:** Chuansheng Wu, Yuyue Li, Lingling Qi

**Affiliations:** 1 School of Economics and Management, Chongqing Jiaotong University, Chongqing 400074, China; 2 School of Management Science and Real Estate, Chongqing University, Chongqing 400044, China

**Keywords:** ecological well-being performance, green transformation, sustainable development, spatial econometrics

## Abstract

The contradiction between the endless pursuit of material possessions and finite natural resources hampers ecological well-being performance (EWP) improvement. Green transformation, recognized as an emerging strategy in sustainable development, can help to coordinate ecological, social, and economic growth by optimizing resource usage, with the ultimate objective of enhancing EWP. This research quantifies how green transformation influences EWP by using panel data from 78 prefecture-level cities in western China from 2012 to 2019. Using the super-SBM and entropy weight models, we assess the EWP and green transformation index (GTI) of 78 prefecture-level cities in western China. On this basis, we quantify the spatial characteristics of EWP by an analysis of the Theil index and spatial autocorrelation. Finally, we examine how GTI affects EWP using the Spatial Durbin model. The results demonstrate that the GTI can raise the EWP of local and nearby cities in western China. According to a GTI analysis of internal indicators, the industrial solid waste usage, harm-less treatment rate of domestic waste, savings level, and R&D expenditure significantly affect EWP. In contrast, the soot emission and consumption levels impede EWP advancement. The analysis of effect decomposition indicates that the sewage treatment rate, expenditure on science and technology, and green patents have a significant spatial spillover effect on the improvement of EWP.

## 1. Introduction

Most cities in developing nations have experienced or are facing the critical challenge of how to shift from rapid development to sustainable development. With the ultimate goal of enriching human well-being, sustainable development places an emphasis on the coordination of ecological sustainability, economic development, and social welfare development [1]. Due to the excessive use of natural resources brought on by the aimless pursuit of rapid economic growth, unsustainability has been negatively affecting well-being in western China on a consistent basis [2]. Western China’s ecosystem has paid a high price for problems such as soil erosion, desertification, and natural disasters, which have suffered irreparable damage or will take an extremely long period to recover [3]. In addition, the western region still lags behind the central and eastern regions, and contains the majority of China’s elderly, borderline, and impoverished areas which are plagued by significant shortages in employment, education, health care, and other livelihood issues [4]. Therefore, western China urgently needs to shift from rapid development to sustainable development.

Achieving higher levels of human well-being with limited natural resources is essential to sustainable development. To assess the extent of sustainable development in each nation, Daly for the first time defined ecological well-being performance (EWP) as the efficiency of transforming natural resource consumption into increased human well-being [5]. According to the green economy theory, this term aims to resolve the “trilemma” of ecological preservation, economic growth, and social welfare improvement that ultimately improves overall human well-being, which provides a new research perspective on sustainable development [6]. Many academics studying the relationship between the environment and human beings argue that sustainable well-being contributes to environmental and human flourishing [7]. Therefore, the implementation of the 2030 Agenda for Sustainable Development will be facilitated by the international community’s action in pressing all nations to improve their ecological well-being performance [8]. To achieve long-term, higher-quality, and more efficient development for people in the western region as a whole, in 2020, the “Guiding Opinions on Promoting the Development of the Western Region in a New Era to Form a New Pattern” was issued by China’s State Council to continue to promote Western Development Strategy (State Council, 2020). However, ecological deterioration and scarcity of natural resources hampered progress in improving EWP in western China. In 2020, areas of poor ecological quality accounted for 31.3 percent of the national total, where harsh conditions limit human survival. Therefore, enhancing ecological well-being performance is necessary for sustainable development and human well-being improvement in the western region [9].

Urban green transformation has become an urgent strategy for sustainable development against the backdrop of ecological civilization. It is considered a viable means to simultaneously achieve high-quality economic growth, environmental conservation, and human well-being improvement, which impacts on ecological well-being performance [10]. Based on green economics, this term is primarily described as the actions aimed at converting outdated production and consumption patterns into green production and living development modes, hence making the growth process more resource efficient and clean [11]. The green transformation has become a global consensus for economic transformation and sustainable development, as it has the potential to improve ecological well-being performance through green production, renewable energy, and low carbon transportation. The Paris Agreement intends to coordinate global efforts to prevent climate deterioration and dramatically reduce greenhouse gas emissions [12]. Additionally, the Green Deal strives to ensure that present and future generations enjoy a high standard of life as an agreement to strike a balance between economic growth and environmental preservation in the EU [13]. Since the carbon neutrality goal was established, China has employed the green transformation as a strategic option in environmental policies to break through the development bottleneck. Previous policies such as the Action Plan for Air Pollution Prevention and Control (State Council, 2013) and the Three-Year Action Plan for Winning the Blue Sky Defense War (State Council, 2018) have successfully alleviated environmental pressure by limiting overall pollution emissions through end-of-pipe treatments. The percentage of good days increased to 87 percent in China in 2020, and the average PM2.5 concentration reduced by 28.8% from 2015. The Work Plan for Ecological Poverty Alleviation (State Council, 2018) has achieved ecological improvement and poverty alleviation in underdeveloped areas. Additionally, it promoted 20 million poor people to increase their incomes, generated more than 52 million employment, accumulated afforestation, and restored 74.5 million mu of grass (National Rural Revitalization Bureau, 2021). To accomplish carbon peaking and further promote the green transformation, the Action Plan for Carbon Peaking by 2030 (State Council, 2021) proposes a comprehensive green transformation.

Therefore, this study aims to quantitatively evaluate how much green transformation contributes to improving ecological well-being performance in China’s western region. We answered the following research questions: (1) What level of development does EWP now have in western cities? (2) What is the current state of the green transformation in western cities? (3) How has the green transformation affected the EWP in western China cities? The authors firstly assess the level of EWP and the state of green transformation development in western China, then investigate the impact of green transformation development on EWP and its spatial effect, and further explore the factors that promote EWP in green transformation development.

## 2. Literature

### 2.1. The Connotation and Assessment of Ecological Well-Being Performance

Due to the scarcity of natural resources, improving ecological well-being performance has emerged as a sustainable development goal for human well-being enhancement and high-quality urban development. The term “Ecological Well-being Performance” (EWP), which provides a new study perspective on sustainable development, is used to assess the efficiency of the transformation of ecological consumption into human well-being based on the principles of the green economy and circular economy [14]. The green economy is an economic growth system that considers environmental protection and resource conservation, emphasizing economic growth within natural resource constraints [15]. In comparison, the circular economy is an economic model that improves the utilization of natural resources by reducing, reusing, recycling, and recovering resources [16]. In line with the connotations of “green economy” and “circular economy”, enhancing the efficiency of converting ecological consumption into well-being can fundamentally reduce natural capital consumption. Daly initially proposed the concept of EWP [5], which pursues the sustainable development dimensions of economic, social well-being, and environmental, in addition to acknowledging the finiteness of natural resources.

The research on EWP focuses on three main aspects: the measurement method, spatial characteristics, and EWP influencing factors. To begin, there are two primary methods for calculating EWP. One is the single ratio method based on the definition. For example, Behjat A. and Tarazkar M. H. defined the ratio of Human Development Index (HDI) to Ecological Footprint (EF) as ecological well-being performance to investigate EWP for Iran from 1994 to 2014 [17]. The improved three-dimension EF and HDI were utilized by Long X., Yu H., Sun M. et al. to evaluate the EWP in four major Chinese island regions in 2017 [18]. The second one is the DEA model to explore the improvement factors of the indicators. For instance, Bian J. et al. studied 278 Chinese cities’ EWP from 2005 to 2016 using the Super-SBM model [19]. The Chinese provinces’ EWP between 2006 and 2017 was calculated by Hou J. et al., using the two-stage super-SBM model [20]. The second method for measuring EWP is more comprehensive and objective than the first. However, the evaluation index system of EWP needs to be further improved. With several scholars discussing the ecological limitations of the HDI, there is now an ecological convergence in measuring sustainable well-being [21]. Second, we investigated the spatial differences in EWP. Wang R. and Feng Y. calculated the Theil Index to investigate the regional differences in EWP in China. It was evident from the intragroup and intergroup differences that China’s EWP decreased steadily from 2006 to 2018 [22]. Wang S. et al. demonstrated that the regional imbalance in China’s green development was continuously worsening from 1997 to 2017, using the Gini Coefficient [23]. Nevertheless, few scholars have researched the spatial correlation of EWP. Third, various scholars have discussed the impact of factors on EWP. Zhou L. and Zhang Z. analyzed the effect of income inequality, technology levels, urbanization levels, and industrial structure on EWP [24]. Hu M. et al. studied the impact of technological innovation, industrialization, government policy, and industrial structure on EWP [25].

### 2.2. The Connotation and Assessment of Green Transformation

The green transformation has emerged as a critical global sustainable development and environmental governance strategy due to increasing environmental pollution. In the context of the green economy and circular economy, this term is considered an action to deal with resource depletion and environmental degradation, thereby improving resource utilization, coordinating environment protection, high-quality economic growth, and social progress [26]. Pearce proposed the concept of the green economy, which was the first time when resources and the environment were connected with economic development. In contrast, previously, economic growth frequently relied on the consumption of natural resources as though these resources were inexhaustible [27]. The circular economy is a system that “as long as possible maintains the value time of products, materials, and resources in economy, and minimizes the waste generation” [28], according to the European Commission. It emphasizes the entire process and outcomes of economic activity. Consequently, green transformation is consistent with the theory of the green economy and circular economy. It not only considers the entire process of economic production but also combines economic production, social development, resources, and the environment, aiming to maximize the performance of resource utilization by transforming the model of economic development [29].

The green transformation can be assessed in two ways. The first is to build a comprehensive assessment framework by calculating the green transformation level using the entropy weight method or analytic hierarchy process. For instance, Wang Y. et al. constructed the city transformation framework system from the perspectives of the economic transformation trend, environmental friendliness trend, and people’s livelihood improvement trend. They used the entropy weight method to investigate the green transformation performance of 115 Chinese cities [30]. To assess the level of green development of 30 coal-resource-based cities in China by using the TOPSIS method, Long R. et al. created an evaluation index system that considers economic green development, social green development, environmental green development, and resource green development [31]. Different assessment index systems have been developed to accurately measure a region or a city’s level of green transformation. Although there is still no unified standard for evaluating green transformation, they all agree about constructing the green transformation evaluation framework from the multiple indicators of the economy, society, and environment. The second one primarily employs non-parametric methods to reflect green transformation. Factors such as green transformation performance and green total factor productivity (GTFP) can resolve issues with multiple inputs and outputs and do not necessitate the establishment of a functional form. For example, Cui H., Liu X., and Zhao Q. used the DEA to calculate 30 Chinese provinces’ provincial green total factor productivity (GTFP) between 2001 and 2017 [32]. Shi D., Xiang W., and Zhang W. applied an improved Data Envelopment Analysis method to explore the level of green development in China from 2011 to 2017 [33]. Green transformation is a comprehensive concept that encompasses ecological, social, and economic transformation. As a result, to effectively assess the green transformation of regions, it is required to construct an evaluation framework system from these aspects.

### 2.3. The Impact of Green Transformation on Ecological Well-Being Performance

According to sustainable development theory, green transformation as a sustainable development strategy influences the efficiency of converting ecological consumption into human well-being. Sustainable development aims to attain the highest degree of development at the lowest possible ecological cost [34], and the impact of green transformation on EWP is mainly through two approaches: reducing input or increasing output. Lowering ecological consumption costs while maintaining social well-being can also enhance EWP. Conversely, it is also feasible to prioritize maximizing human well-being as the eventual consequence of economic activities.

The studies on various indicators and approaches to green transformation provide a theoretical foundation for exploring the impact of green transformation on ecological well-being performance. For instance, Cao Y. and Bian Y. indicated that ecological environmental performance is significantly influenced by the rate of harmless treatment of household waste, environmental protection funds, and PM2.5 [35]. Zhang H. et al. demonstrated that natural resource rent, green innovation, and green investment significantly improved the ecological footprint performance [36]. Spatial spillover effects have also been discussed in this field to explore the mechanism underlying green transformation and ecological well-being performance. Based on the spatial econometrics model, Feng Y. et al. demonstrated that green total factor productivity (GTFP) and spatial spillover are the primary drivers for EWP improvement in Chinese provinces [37]. Zhao X., Shang Y., and Song M. revealed that the green industry could enhance ecological efficiency using the spatial lag model. However, the high reliance on natural resources will inhibit this effect [38].

In conclusion, numerous academics have conducted in-depth studies on the impact of green innovation, green finance, green total factor productivity, and green industrial output on ecological well-being performance, providing a theoretical foundation and methodology reference. However, from the perspective of urban green transformation, no studies have been systematically conducted on the effect of green transformation on EWP.

### 2.4. Research Review

In conclusion, the literature has three gaps. First, there was insufficient research on the comprehensive EWP index system. Ecological well-being performance encompasses the aspects of the economic, social, and ecological well-being. Input elements need to cover human and social capital to achieve resource utilization, and well-being output needs to increase related ecological well-being. Second, we further need to quantitatively analyze EWP’s spatial characteristics. Few scholars have investigated the spatial correlation analysis of EWP between regions, as previous studies primarily focused on regional differences in EWP. The agglomeration’s spatial correlations and geographical differences can influence EWP, which is a complicated and open system. Third, the impact analysis of green transformation on EWP generally involves only individual dimensions and single indicators. Despite the fact that urban green transformation is a diverse and ever-evolving process, the existing studies lack systematic research on this matter in the literature.

**Figure 1:**
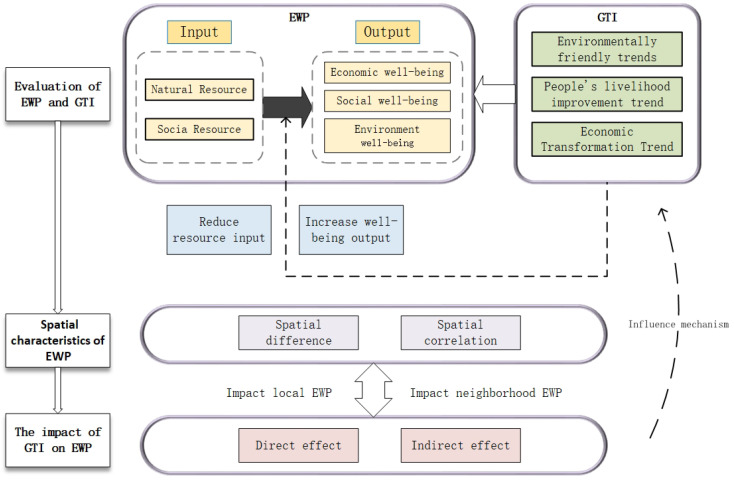
The research framework for the impact of green transition on ecological welfare performance.

We therefore propose the research framework for this paper following a research review and an explanation of the research questions. As shown in Figure 1, we aim to explore whether and how green transformation affects ecological well-being performance and the influencing mechanisms and regional heterogeneity. We seek to investigate the development situation and spatial characteristics of EWP in western China and further to quantitatively assess the impact of green transformation on EWP. The research offers a decision-making reference for improving EWP and green transformation development in western Chinese cities, which has theoretical and practical implications for sustainable urban development and enriches the relevant research on ecological well-being performance.

## 3. Methods

### 3.1. Research Area

**Figure 2:**
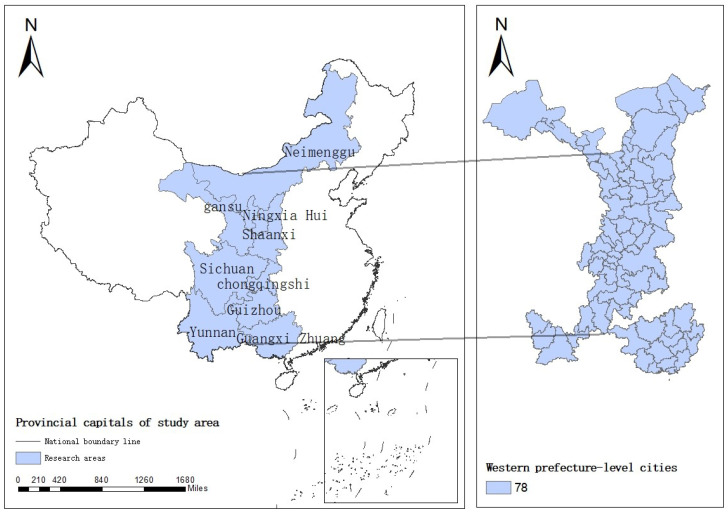
Research areas.

This study focuses on western China, which makes up 72 percent of China’s total landmass and spans an area of over 686 km2. As shown on the left of Figure 2, it consists of 12 provinces: Sichuan, Chongqing, Yunnan, Guizhou, Tibet, Shaanxi, Gansu, Ningxia, Qinghai, Xinjiang, Inner Mongolia, and Guangxi. Western China is rich in minerals, water, land, and other resources. However, due to topographic constraints, the western part of China, especially the northwest, is backward in terms of economic development and poor in climate conditions. For example, the quality of land resources in the western regions is quite different from that in the eastern and central regions, which cannot grow food on a large scale. As shown on the right in Figure 2, the final research subject consisted of 78 prefecture cities in 9 provinces in western China due to the incomplete data and the differences in statistical caliber of Xinjiang and Qinghai.

### 3.2. Data Source

This section measures the EWP and GTI of 78 cities in western China from 2012 to 2019, then evaluates the spatial characteristics of EWP and the effect of GTI on EWP. The primary sources of the research data are the China City Statistical Yearbook (2013–2020), China Urban Construction Statistical Yearbook (2013–2020), Statistical Bulletin of the National Economic and Social Development (2013–2020), and the statistical data of municipal government departments. We calculated and collated the indicators at the per capita and proportion level to eliminate the impact of city size. The individual missing values were supplemented by the interpolation method to strive for the integrity and accuracy of the data.

### 3.3. Ecological Well-Being Performance Evaluation

#### 3.3.1. Ecological Well-Being Performance Framework System

As ecological well-being performance can broadly be defined as transforming the efficiency of resource consumption into increased human well-being [39], this paper constructs the analysis framework of EWP from the perspective of input–output. Table 1 displays the studies related to ecological well-being performance and focuses on the selection of indicators in each piece of literature. In this paper, the input indicators included natural and non-natural resources, and the output indicators included economic, social, and ecological well-being. Previous studies showed that water, land, and energy are the natural resources on which human beings depend for survival [19,23,24]. Besides, labor and capital, which are essential intermediate means to achieve resource utilization, were suggested as input variables by some scholars [23,40]. On the other hand, the human development index is the most widely used indicator to assess the well-being of countries and regions, with GDP, education, and health represented to reflect economic well-being and social well-being [8,17,18]. Numerous academics discussed the ecological limitations of HDI. Well-being frameworks need to consider the environment or the interrelationships between people and their environment [21,41]. Therefore, we choose per capita rate of parks and green areas as ecological well-being indicators in this paper [25,42]. In addition, due to the incomplete of prefecture-level cities’ data with regard to the life expectancy, we select the number of physicians per 1000 people to substitute [18,43]. Table 2 displays the main characteristics of the input–output indicators.

#### 3.3.2. Ecological Well-Being Performance Calculation

We constructed an input–output assessment system based on the framework system for ecological well-being performance. Therefore, the super-SBM model was employed to calculate the EWP with multiple inputs and outputs of cities in western China. The Super-SBM model, proposed by Tone [44], was frequently used to measure EWP owing to eliminating subjectivity and minute errors. Assume there are *n* cities, *m* kinds of input elements, and *q* types of output elements. The super-SBM model is defined as follows:

(1) $$\rho^{*}=min\frac{\frac{1}{m}\sum_{i=1}^{m}\left(\frac{s^{-}}{x_{ik}}\right)}{\frac{1}{q}\left(\sum_{r=1}^{q}\frac{\bar{s^{+}}}{y_{rk}}\right)}$$

(2) $$x_{ik}\geq \overset{n}{\underset{j=1,\;\;\;\neq k}{\sum }}x_{ij}\lambda_{j}-s_{i}^{-}\;\left(i=1,2,\ldots ,m\right)$$

(3) $$y_{rk}\leq \overset{n}{\underset{j=1,\;\;\;\neq k}{\sum }}y_{rj}\lambda_{j}+s_{r}^{+}\;\left(r=1,2,\ldots ,q\right)$$

(4) $$\lambda_{j},\;s_{i}^{-},\;s_{r}^{+}\geq 0$$

(5) j=1,2,…,n;i=1,2,…,m;r=1,2,…,q

where $\rho^{*}$ is the target ecological well-being performance; $s_{i}^{-}$ and $s_{r}^{+}$ are slack variables of input and output; and $\lambda_{j}$ is the weight of the $j^{th}$ DMU’s input and output.

### 3.4. Green Transformation Evaluation

#### 3.4.1. Green Transformation Framework System

Green transformation can be defined as a comprehensive concept which is concerned with economic, social, and ecological transformation. As a result, this paper proposes an evaluating framework for the green transformation index (GTI) from these aspects: economic transformation trend, environmentally friendly trend, and people’s livelihood improvement trend. It considers the connotations, relevant national programming, and related studies about green transformation. The framework emphasizes the dynamic measurement process of green transformation: improve quality and efficiency and reasonable innovation, which requires a steady increase in the economy and well-being, and environmental protection. Table 3 displays the main characteristics of the 13 indicators. The environmentally friendly trend (EFT) includes environmental treatment and pollution [15,33,45]. The people’s livelihood improvement trend (PIT) comprehensively assesses the green and low-carbon lifestyle trend for residents in terms of work and employee payments, and residents’ lives [30,46]. The economic transformation trend (ETT) comprehensively assesses the trend of green production through technological development [28,47,48].

#### 3.4.2. Green Transformation Index Calculation

Based on the framework of green transformation, we constructed the GTI using the entropy weight method, which considers each dimension’s contribution. The entropy weight method provides the basis for the comprehensive system of multiple indicators and can effectively avoid subjective judgement errors [49]. Following the procedures, we measured the GTI:

Step 1: Structure evaluation matrix X. According to the evaluation objects and indicators of GTI, set the $j_{th}$ indicator of the $i_{th}$ object is $X_{ij}$:

(6) $$X=\;{\left[\begin{array}{ccc} X_{11} & \cdots & X_{1n}\\ \vdots & \ddots & \vdots \\ X_{m1} & \cdots & X_{mn} \end{array}\right]}_{mxn}$$

(7) I=1, 2, …, m; j=1, 2, …, n 

Step 2: Normalize the matrix X. Due to the different quantitative standards of each indicator, the indicator data need to be dimensionless:

(8) $$\mathrm{Positive}\;\mathrm{indicators}:\;x_{ij}=\frac{X_{ij}-min\left(X_{j}\right)}{max\left(X_{j}\right)-min\left(X_{j}\right)}$$

(9) $$\mathrm{Negative}\;\mathrm{indicators}:\;x_{ij}=\frac{max\left(X_{j}\right)-X_{ij}}{max\left(X_{j}\right)-min\left(X_{j}\right)}$$

Step 3: Calculate the proportion $P_{ij}$ of each indicator:

(10) $$P_{ij}=\frac{x_{ij}}{\sum_{i=1}^{m}x_{ij}}$$

Step 4: Calculate each indicator’s information entropy $E_{j}$ and information utility $D_{j}$:

(11) $$E_{j}=-{\left(In\left(m\right)\right)}^{-1}\overset{m}{\underset{i=1}{\sum }}P_{ij}In\left(P_{ij}\right)$$

(12) $$D_{j}=1-E_{j}$$

Step 5: Calculate each indicator’s entropy weight $W_{ij}$ of:

(13) $$W_{j}=\frac{D_{j}}{\sum_{j=1}^{n}D_{j}}$$

Step 6: Calculate the $GIT_{i}$ of the $i_{th}$ city in western China:

(14) $$GTI_{i}=\overset{n}{\underset{j}{\sum }}W_{j}x_{ij}$$

### 3.5. Analysis of EWP’s Spatial Differences and Spatial Correlation

#### 3.5.1. Measure the Spatial Differences of EWP

To quantitatively analyze the spatial differences of the EWP of western Chinese cities, we investigated the regional differences and sources using the Theil index. Theil proposed the Theil index to measure regional inequality, which is decomposed into intra-differences and inter-differences between the regions to analyze the different characteristics of the region [50]. Calculate the Theil index as follows:

(15) $$\mathrm{Theil}=\frac{1}{n}\overset{n}{\underset{i=1}{\sum }}\frac{y_{i}}{\bar{y}}In\left(\frac{y_{i}}{\bar{y}}\right)$$

where $y_{i}$ is the EWP of the $i_{th}$ city. Assume *n* cities are divided into K groups, each of which is represented by the expression $g_{k}$(*k* = 1, …, *K*). Since $n_{k}$ belongs to the $k_{th}$ group, we get $\overset{K}{\underset{k=1}{\sum }}n_{k}=n$. The $T_{b}$ and $T_{w}$ are the difference between groups and the difference within groups, the Thiel index can be decomposed as follows:

(16) $$\mathrm{Theil}=T_{b}+T_{w}=\overset{k}{\underset{k=1}{\sum }}Y_{k}In\left(\frac{Y_{k}}{n_{k}/n}\right)+\overset{k}{\underset{k=1}{\sum }}Y_{k}\left(\underset{i\in g_{k}}{\sum }\frac{Y_{i}}{Y_{k}}In\left(\frac{Y_{i}/Y_{k}}{1/n_{k}}\right)\right)$$

where $Y_{i}$ is the proportion of the $i_{th}$ city; $Y_{k}$ is the proportion of the $k_{th}$ group.

(17) $$T_{k}=\underset{i\in g_{k}}{\sum }\frac{Y_{i}}{Y_{k}}In\left(\frac{Y_{i}/Y_{k}}{1/n_{k}}\right)$$

(18) $$D_{k}=Y_{k}\frac{T_{k}}{T},\;D_{b}=\frac{T_{b}}{T}$$

where $T_{k}$ is the difference within the $k_{th}$ group; $D_{k}$ and $D_{b}$ is the contribution rate between groups and the contribution rate within groups $k_{th}$.

#### 3.5.2. Measure the Spatial Difference of EWP

The first law of geography of Tobler, which states that geographical elements have a mutual dependence on one another, is represented by spatial autocorrelation [51]. To further explore the spatial correlation characteristics of EWP between cities, we employed the global Moran’s I index to determine if a specific attribute shows an aggregation or dispersion trend in the regions. The global Moran’s I is calculated as follows:

(19) $$I=\frac{n\sum_{i=1}^{n}\sum_{j=1}^{n}w_{ij}\left(x_{i}-\bar{x}\right)\left(x_{j}-\bar{x}\right)}{\sum_{i=1}^{n}\sum_{j=1}^{n}w_{ij}\sum_{i=1}^{n}{\left(x_{i}-\bar{x}\right)}^{2}}$$

where $x_{i}$ and $x_{j}$ are the EWP of the $i_{th}$ and $j_{th};$ $w_{ij}$ is the spatial weight, which indicates the spatial proximity between the cities *i* and *j*. If *i* is adjacent to *j*, $w_{ij}$ = 1, otherwise $w_{ij}$ = 0. The Moran’s I index ranges from −1 to 1. I > 0 denotes a positive spatial correlation, I < 0 denotes a negative spatial correlation, and I = 0 denotes spatial uncorrelation. The local trend is observed using the Local Indicators of Spatial Association (LISA) method:

(20) $$I=\frac{\left(x_{i}-\bar{x}\right)}{S^{2}}\overset{n}{\underset{i=1}{\sum }}\left(x_{i}-\bar{x}\right)$$

### 3.6. Analysis of EWP’s Spatial Differences and Spatial Correlation

After measuring the results of EWP and GTI in the western cities, we employed spatial econometric models to investigate the impact of GTI on EWP and the effect mechanism of each GTI dimension on EWP. In this research, we included all of the GTI indicators in the framework that manifested how the internal structure of green transformation affected the EWP. The explained variable is the ecological well-being performance (EWP), and the core explanatory variable is the green transformation index and the variables at the index level. Among the explanatory variables, EFT, PIT, and ETT represent the environmentally friendly trend, people’s livelihood improvement trend, and economic transformation trend, respectively. ISW, ST, DW, SD, SO2, IS, UP, EW, HS, CS, RD, SC, and GP, respectively, are the X1 to X13 of GTI, as Table A1 in the Appendix A for details.

The Spatial Lag Model (SLM), Spatial Error Model (SEM), and Spatial Dobbin Model (SDM) are frequently applied to describe spatial correlation [52].

The ordinary least squares model is expressed as:

(21) $${\mathrm{EWP}}_{it}=\alpha_{i}+\beta_{1}X_{it}+\varepsilon_{it}+\mu_{i}+\upsilon_{t}$$

The SLM assumes that urban ecological well-being performance is affected by neighboring cities, due to the spatial spillover effect. The SLM is formed as:

(22) $${\mathrm{EWP}}_{it}=\alpha_{i}+\rho W{\mathrm{EWP}}_{it}+\beta X_{it}+\varepsilon_{it}+\mu_{i}+\upsilon_{t}$$

Through the spatial dependency among the error terms, which are linked to both the nearby and local cities, the SEM takes into account the spatial relationships:

(23) $${\mathrm{EWP}}_{it}=\alpha_{i}+\beta X_{it}+\varepsilon_{it}+\mu_{i}+\upsilon_{t}$$

(24) $$\varepsilon_{it}=\lambda W_{\varepsilon t}+\varphi_{it}$$

The SDM combines the characteristics of the SLM and SEM:

(25) $${\mathrm{EWP}}_{it}=\alpha_{i}+\rho WEWP_{it}+\beta X_{it}+\theta WX_{it}+\varepsilon_{it}+\mu_{i}+\upsilon_{t}$$

where $\alpha_{i}$ is an intercept term; $\varepsilon_{it}$ is an error term; $\beta_{i}$ are parameters to be estimated, $\mu_{i}$ is a space fixed effect; $\upsilon_{t}$ is a time fixed effect; ρ is a spatial spillover coefficient; λ is the spatial coefficient of the error term; *W* is a spatial weight matrix; $\varphi_{it}$ is a random error; ${\mathrm{EWP}}_{it}$ is the explained variable; and $X_{it}$ is the explanatory variable.

## 4. Results

### 4.1. Ecological Well-Being Performance of Western Cities

**Figure 3:**
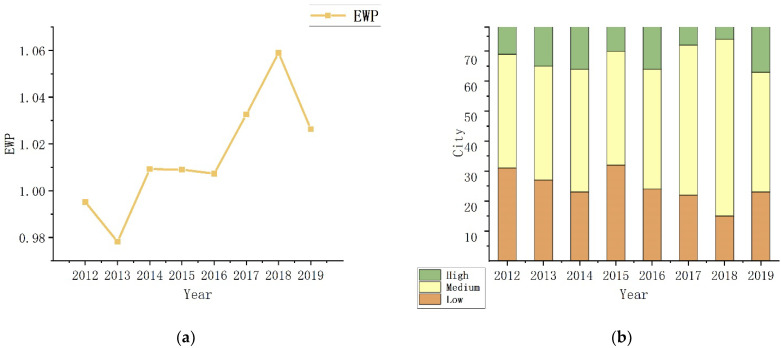
(**a**) Average EWP 2012–2019; (**b**) Classification of EWP 2012–2019.

Figure 3a depicts the trend of the annual evolution of the average value of EWP, showing an oscillating upward tendency and a medium level from 0.9951 in 2012 to 1.0263 in 2019. The adoption of pertinent national policies has resulted in an effectively improved EWP. However, there are still significant differences and polarization between cities. Jiayuguan (1.5876), Ordos (1.4628), and Zhaotong (1.4258) are the top three out of the 78 cities, while Qinzhou (0.5995), Anshun (0.6188), and Luzhou (0.6263) are the bottom three. In addition, 44.87 percent of cities failed to achieve DEA effectiveness during the entire surveyed period based on the annual value for each city, demonstrating the low efficiency of many of the western cities in improving their overall ecological well-being performance. According to research of EWP’s time evolution, increasing policy support will further enhances these cities’ capabilities for sustainable development. As illustrated in Figure 3b, the 78 sample cities are divided into three groups: high efficiency, medium efficiency, and low efficiency using the natural interval classification method. Overall, the majority of cities belong to the medium efficiency and low efficiency groups. The low efficiency cities are gradually evolving into medium efficiency ones from the perspective of the temporal development tendency. The low efficiency cities are more effective at improving EWP than the high efficiency cities, as shown by the number of low efficiency cities that decreased from 31 (39.74 percent) to 23 (29.49 percent), while the number of high efficiency cities increased from 9 (11.54 percent) to 15 (19.23 percent) from 2012 to 2019. Accordingly, the studies contend that raising the efficiency of the low efficiency cities is essential to raising EWP overall.

### 4.2. Green Transformation Index of Western Cities

**Figure 4:**
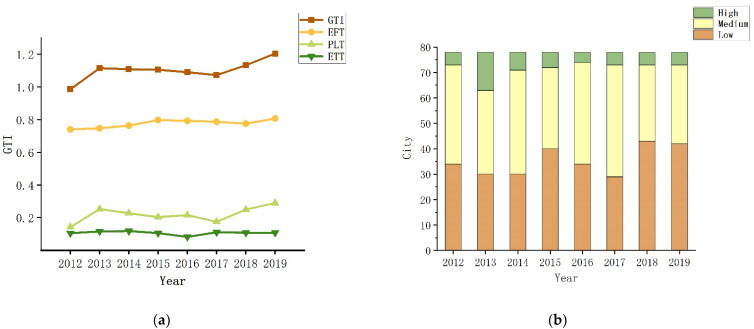
(**a**) Average GTI, FFT, PLT, ETT 2012–2019; (**b**) Classification of GTI 2012–2019.

Figure 4a displays the overall trend of the GTI, EFT, PLT, and ETT. The green transformation index in western cities showed a slight improvement and steady progress in green transformation of the western cities, with the average value increasing from 0.9856 in 2012 to 1.2035 in 2019. Nevertheless, the GTI growth rate was slow since it would be hard to correctly resolve, brought on by this protracted period of development in the short term. From each dimension of the GTI, the environmentally friendly trend (EFT) increased from 0.7397 to 0.8067, the people’s livelihood improvement trend (PLT) increased from 0.1415 to 0.2891, the economic transformation trend (ETT) increased from 0.1044 to 0.1077, with a mean contribution of 70.44 percent, 19.9 percent, and 9.66 percent for EFT, PLT, and ETT, respectively. The outcome demonstrates that the environmentally friendly trend has developed to a stabilized level that was relatively high compared to other dimensions. However, the trend for people’s livelihood improvement and economic transformation is less favorable in the western region and needs to be accelerated. As shown in Figure 4b, the 78 sample cities are classified into three groups based on their GTI: high level, medium level, and low level, indicating that most of the cities had low- and medium-level green transformation development. In comparison, the high-level cities are usually economically developed regions where GTI is well ahead of other cities, such as Chengdu (2.1234), Xian (2.0015), Erdos (1.8674), and Chongqing (1.6302). Between 2012 and 2019, the number of low-level cities decreased from 39 to 31. In contrast, the number of medium-level cities increased from 32 to 39, showing that it is more difficult for medium and low-level cities to develop green transformation than high-level cities. The cities with low-level GTI need to focus more attention on future policies.

### 4.3. Spatial Characters of EWP in Western Cities

We used the Theil index and spatial autocorrelation to quantitatively analyze spatial characters of EWP in order to more precisely identify the degree of the close gap in the western cities.

#### 4.3.1. Measure the Spatial Difference of EWP

**Figure 5:**
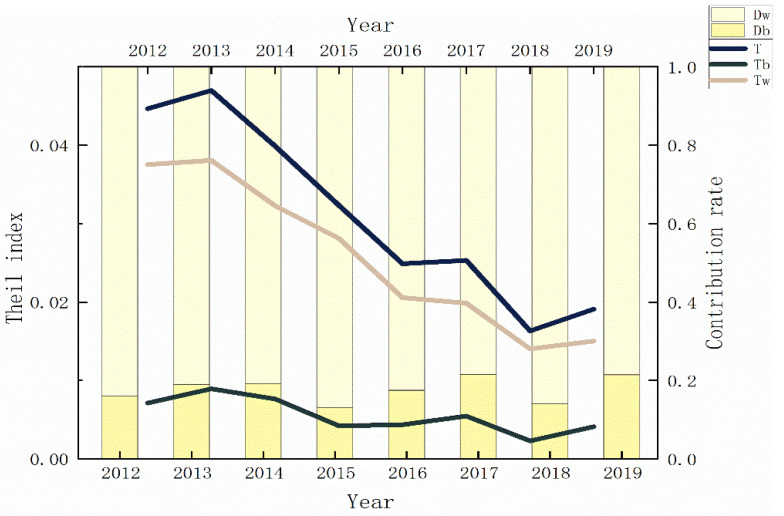
Theil index and regional contribution rate 2012–2019.

We divided the 78 cities into 9 groups and measured the Theil index by Stata to investigate the spatial difference between the cities’ EWP. Figure 5 displays the Theil index results, indicating that the spatial difference of EWP exhibited a steadily downward trend between 2012 and 2019. As shown in Figure 5, the evolutionary tendency of intra-differences is comparable with that of the overall regional difference. The inter-differences were relatively stable with a small change range. Specifically, the total regional difference reduced from 0.0446 to 0.0191, the intra-differences decreased from 0.0375 to 0.015, and the inter-differences declined from 0.0071 to 0.0041. It indicates that the overall spatial differences of EWP in western China have decreased significantly as has the regional imbalance. Regarding to the contribution of different sources, the average intra-differences’ contribution to the total contribution is 80%, indicating that intra-differences are the primary factor influencing the overall difference. Therefore, reducing the intra-differences is essential to narrowing the regional gap in EWP. The government need to aggressively adopt measures to coordinate regional development while considering the differences.

#### 4.3.2. Spatial Correlation of EWP

Using ArcGIS to explore the spatial correlation of 78 cities’ EWP, we measured the global and local spatial autocorrelation statistics. The Moran’s I fluctuated from 0.145 to 0.224, as shown in Table 4, revealing that there is a considerable positive spatial autocorrelation of EWP between cities, though it failed to meet the threshold significance in 2018 and 2019. Therefore, the ecological well-being performance in the western cities had a specific agglomeration effect, each city’s EWP was affected by its input–output system and nearby cities’ EWP.

**Figure 6:**
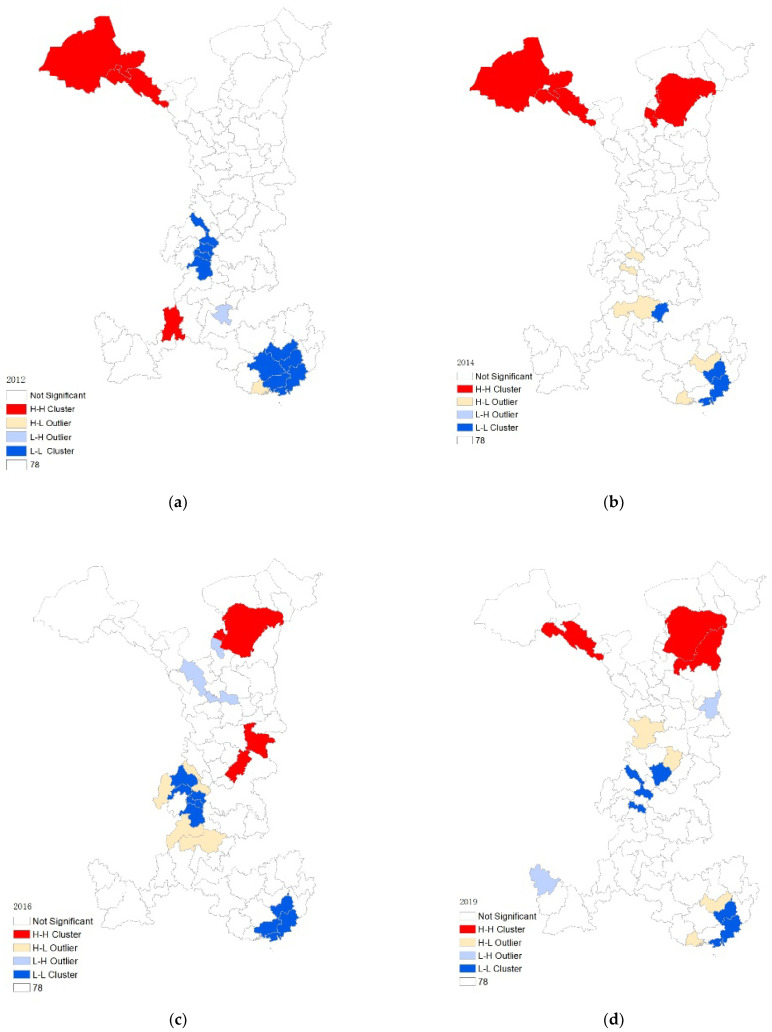
(**a**) 2012 LISA clustering chart; (**b**) 2014 LISA clustering chart (**c**) 2016 LISA clustering chart; (**d**) 2019 LISA clustering chart.

Figure 6 displays the LISA agglomeration plots for 2012, 2014, 2016, and 2019, revealing the local spatial relationship of EWP in the western cities. We classify the spatial agglomeration into four groups: (1) H-H agglomeration areas. The spatial difference of such agglomeration regions is minor, with relatively high EWP values for the cities themselves and their surrounding cities. It is mainly distributed in Jiuquan, Jialingguan, Zhangye as the center of Gansu, Kunming, Qujing, Pul as the center of Yunnan, Huhhot, and Wuzhong as the center of the HuBaoE city group. (2) H-L agglomeration areas. The cities in these areas have high EWP values on their own but are surrounded by cities with low EWP values, reflecting high-value anomalies. (3) L-H agglomeration areas. The cities in these areas have low EWP values on their own but are surrounded by cities with high EWP values, which reflects low-value anomalies. (4) L-L agglomeration areas. In such areas, the EWP values of the city itself and its nearby cities are relatively low, and there are not many spatial differences in size between the agglomeration areas. It is primarily distributed in Guangxi with Beihai, Yulin, and Guigang as the center and Sichuan with Zigong, Neijiang, and Leshan as the center.

### 4.4. The Effect of GTI on EWP

The Moran’s I test results demonstrated that spatial attributes are the key elements influencing the EWP of the western cities. As a result, we applied spatial econometric models to explore the impact of GTI on EWP in western China from 2012 to 2019. Table 5 shows the spatial regression results that pass the corresponding tests for the validity of the spatial econometric model. Firstly, the results of the LM spatial lag test (*p* = 0.000), R-LM spatial lag test (*p* = 0.009), LM spatial error test (*p* = 0.000), and R-LM spatial error test (*p* = 0.000) were significant, demonstrating the spatial econometric model outperforms simple regression. Secondly, the Wald and likelihood ratio (LR) test disproved the null hypothesis, indicating that the SDM cannot degenerate into SLM or SEM. Further, the Hausman test results show that the time-fixed effect outperforms the random effect. We eventually decided to investigate the spatial effect of GTI on EWP using the spatial Durbin model with time-fixed effects after merging the regression outcomes of each mode. Green transformation is a complex and comprehensive concept. To further explore the internal mechanism of urban green transformation on EWP, the internal variables of GTI are regressed shown in Table 6. In addition, Table 7 displays the spatial spillover effect decomposition outcomes: direct effect, indirect effect, and total effect.

## 5. Discussion

The findings of this study suggest that the green transformation index significantly improves ecological well-being performance in terms of spatial spillover effects. As summarized in Table 4 and Table 5, the variables in each model have the same values, indicating the reliability of the findings. The spatial lag coefficient in the SDM model is significantly positive at the 10% level, indicating a favorable spatial spillover effect between the EWP of nearby regions. Every percent increase in a city’s EWP could lead to a corresponding increase of 0.093 percent in nearby cities. The findings demonstrate that EWP growth is impacted by the input and output elements of the cities themselves as well as the spatial spillover effect of neighboring cities, following the previous findings of the spatial autocorrelation test.

First of all, EFT significantly affects the improvement of EWP. Under the dimension of the environmentally friendly trend, the comprehensive rate of industrial solid waste utilization and harmless disposal rate of domestic waste are both considerably positive at the 1% level, and the sewage treatment centralized rate is significantly positive at the 10% level. From the standpoint of effect decomposition, the centralized sewage treatment rate has a notably beneficial spillover effect on the adjacent area, with every 1 percent rise in it raising the local EWP by 3.298%, and the EWP of adjacent cities by 6.96%. The results demonstrate that environmental governance can significantly improve EWP, requiring governmental oversight and guidance. The mandatory environmental regulation policy can effectively enhance the level of ecological efficiency by reducing the detrimental effects of environmental pollution and indirectly by lowering resource reliance [53]. On the other hand, the soot emissions are extremely harmful to human health, which has a negative impact on EWP, with every 1% increase in emissions, it has the effect of decreasing EWP by 16.868%. Since so many pollutants are created in tandem with economic expansion, many cities fall short of meeting the standard set by air quality regulations. In agreement with other studies, reducing the negative spatial spillover effects of environmental pollution would enhance these cities’ efficiency levels [54]. Even as the scale and proportion of investment in environmental governance expand, the pollution emissions at the point of production need to be reduced [55]. To achieve green transformation, the development of ecological civilization has entered a new phase with carbon reduction as the primary aim, which has replaced the previous end treatment with source control, process optimization, end treatment, and waste recycling (National Development and Reform Commission, 2021).

Besides, PIT has a great positive impact on the enhancement of EWP. Under the dimension of the people’s livelihood improvement trend, the household savings and the employees’ wages are statistically positive at the 1 percent level and 5 percent level, while the unemployed have a negative but not significant impact on EWP, indicating that the increase in residents’ income and savings has effectively promoted the improvement of urban EWP. People’s pursuit of education and health are influenced by household savings and income, and households with varying income levels also exhibit noticeably different spending patterns [56]. For example, families with high savings can easily transform to a cleaner lifestyle and consumption model, in contrast to houses with low savings for whom it is challenging to obtain clean energy. However, the consumption level has a 10 percent adverse influence on EWP, which may be related to residents’ lifestyle, behavior habits, and environmental consciousness. Household consumption activities and habits have a substantial effect on resources and the environment. Therefore, it is vital to guide the public to participate in environmental governance and improve citizens’ environmental consciousness to reduce emissions on the consumption side [57]. Public participation is evolving as a new driver for green transformation-related issues.

Finally, ETT has a considerable positive impact in improving EWP. Under the dimension of the economic transformation trend, the R&D expenditure is notably positive at the 1 percent level, indicating that the R&D investment scale is an important driver for EWP. Through technological development and green innovation, economic transformation can not only alter present production and consumption patterns to boost productivity and cut input costs, but also realize cleaner production and lower pollution emissions, which can achieve improved EWP [58]. Additionally, green patents have a spatial spillover effect on EWP that is significant at the 10 percent level. An increase of 1 percent in a city’s green patents can result in a 3.097 percent increase in the region’s EWP and a 14.981 percent increase in the EWP of surrounding areas, indicating the aggregation level of green innovation can remarkably promote the EWP of both local and nearby cities. Green innovation, as opposed to conventional innovation, emerges as characteristic of green development and is innovation driven, enabling access to more environmentally friendly production and the usage of cleaner energy to replace fossil fuels, which can lessen reliance on exhaustible resources and curb carbon emissions [59]. Green technologies or technological innovation is an important driver for green economic growth, which will accelerate the trend of the city’s economic transformation, thus further improving the EWP.

## 6. Conclusions

Based on an analysis of the EWP’s temporal and spatial pattern, we find that EWP improvements in low-efficiency cities are more conducive to overall efficiency improvements. On the other hand, the green transition process of high-efficiency cities is much faster than that of other cities. We demonstrate that green transformation can improve local EWP and the surrounding cities through the spatial spillover effect in western China by systematically investigating the effect of green transformation on ecological well-being performance. By further analyzing the impact mechanism of internal factors of GTI on EWP, we reveal that the rate of industrial solid waste utilization, harmless treatment rate of domestic waste, sewage treatment rate, savings levels, and R&D expenditure significantly affect EWP improvement. In contrast, the soot emissions and consumption levels had an apparent adverse impact on EWP improvement. Finally, the spatial spillover effect decomposition indicates that the sewage treatment rate, the expenditure on science and technology, and green patents had a significant spatial spillover effect on western Chinese cities’ EWP growth.

We propose the following policy recommendations based on the aforementioned research findings. (1) It is essential to vary policy actions to improve EWP in light of the various natural resources and development levels of various cities. The lower efficiency EWP cities need to increase environmental management efforts to address the negative impact of pollutant emissions on the ecological environment. On the other hand, cities with a higher EWP efficiency should take the lead in the more costly green economic transformation in addition to solving the pollution problems [60]. They should fully utilize the spatial spillover effects of environmental governance, technological development, and green technologies to directly and indirectly improve the ecological welfare performance of local and surrounding cities [54], break the regional administrative barriers, and promote the coordinated development of inter-city linkages. (2) All cities should attach importance to people’s livelihood improvement trends. The Sustainable Development Goals (SDGs) call for governments to lessen socioeconomic inequality and enhance the welfare of their citizens to achieve a world of peace, dignity, and prosperity for everyone. On the other side, it is equally crucial to raise public environmental awareness and encourage public participation in environmental governance. Everyone is responsible for practicing green and low-carbon lifestyles and promoting green transformation to enhance ecological well-being performance.

In conclusion, this paper supports a new perspective on sustainable development for western cities, but there are still some limitations. The county and rural areas can be used as research subjects to further investigate the cross-regional differences and influencing elements of EWP.

## Figures and Tables

**Table 1:** Studies related to ecological well-being performance.

Researcher	Method	Input Indicators	Output Indicators
Zhang S. et al. (2018) [6]	Ratio method	Ecological footprint	HDI
Behjat A. et al. (2021) [17]	Ratio method	Ecological footprint	HDI
Long X. et al. (2020) [18]	Ratio method	3D Ecological footprint	HDI
Bian J. et al. (2020) [19]	Super-SBM	Water, land, energy, environmental pollutants	HDI
Hou J. et al. (2020) [20]	Two-stage Super-SBM	Water, land, energy, capital	Economic well-being, social well-being, environmental well-being and pollution
Zhou L. et al. (2021) [23]	Super-SBM	Water, land, energy	GDP, environmental pollution
Hu M. et al. (2021) [24]	Network DEA	Water, land, energy	Economic well-being, social well-being, environmental well-being
Wang S. et al. (2021) [41]	Super-SBM	Water, land, energy, capital, labor	Economic well-being, social well-being, environmental well-being and pollution
Wang R. and Feng Y. (2020) [42]	Super-SBM	Water, land, energy, environmental pollution	HDI
Bian J. et al. (2020) [43]	Super-SBM	Water, energy, capital, labor	HDI, environmental pollution

**Table 2:** Ecological well-being performance measurement index system.

Category	Primary Indicators	Secondary Indicators	Tertiary Indicators
Input	Natural resource	Water	Per capita water consumption
		Land	Per capita urban construction land
		Energy	Energy consumption per 10,000 yuan output value
	Non-natural Resource	labor	Employed persons
		Capital	Per capita investment in fixed assets
Output	Economic well-being	Income	Per capita gross regional product
	Social well-being	Education	Expected years of education
		Health	Physicians per 1000 people
	Ecological well-being	Park	Per capita parks, green areas
		Green	The green coverage rate of built-up area

**Table 3:** Green transformation performance measurement indicator system.

Target Layer	Criteria Layer	Index Layer	Direction
Environmentally friendly trend	Environmental governance	Comprehensive utilization rate of industrial solids	+
		Centralized sewage treatment rate	+
		Harmless disposal rate of domestic waste	+
	Pollutant emissions	Industrial sewage discharge per 10,000 yuan of emissions	−
		Industrial SO2 per 10,000 yuan of output value	−
		Industrial soot emissions per ten thousand yuan of output	−
People’s livelihood improvement trend	Employment improvement	Registered unemployed persons in urban areas	+
		Average wage of employed employees	+
	Residents lives	Per capita household deposit savings	+
		Total retail sales of consumer goods per capita	+
Economic transformation trend	Technological development	R&D expenditures	+
		Ratio of science and technology expenditure to fiscal expenditure	+
		Number of green patent applications per 10,000 people	+

**Table 4:** Global Moran’s I of ecological well-being performance.

Year	Moran’s I	Year	Moran’s I
2012	0.177 ***	2016	0.164 **
2013	0.134 **	2017	0.145 **
2014	0.134 **	2018	−0.067
2015	0.058 *	2019	−0.014

Note: ***, **, * means that the statistics are significant at the levels of 1%, 5%, and 10%, respectively.

**Table 5:** Regression analysis results of the effect of GTI on EWP.

Variable	OSL	SEM	SLM	SDM
EFT	0.498 ***	0.489 ***	0.479 ***	0.474 ***
PIT	0.230 *	0.144	0.178	0.073
ETT	−0.023	0.010	−0.001	0.071
W × EFT				0.022
W × PIT				0.618 ***
W × ETT				−0.179
ρ			0.146 ***	0.093 *
λ		0.143 ***		
$R^{2}$	0.058	0.057	0.060	0.083
Wald spatial lag				7.13 ***
LR spatial lag				13.09 ***
Wald spatial error				14.43 ***
LR spatial error				14.45 ***
Hausman test				15.54 **

Note: ***, **, * means that the statistics are significant at the levels of 1%, 5%, and 10%, respectively.

**Table 6:** Regression results of the internal structure of the impact of GTI on EWP.

Variable	OSL	SEM	SLM	SDM
ISW	1.580 ***	1.589 ***	1.534 ***	1.277 ***
ST	−0.863	3.608 **	3.390 *	3.448 *
DW	0.445 ***	0.463 ***	0.465 ***	0.565 ***
SD	−0.354	−0.499 *	−0.467 *	−0.285
SO2	0.172	−0.129	−0.154	−0.149
IS	−9.568 ***	−20.597 ***	−20.057 ***	−16.868 ***
UP	−0.345	−0.397	−0.412	−0.366
EW	0.511 **	0.665 **	0.625 **	0.495
HS	2.070 ***	1.474 **	1.488 **	1.726 ***
CS	−1.030 **	−0.653	−0.682	−0.823 *
RD	0.323 **	0.324 **	0.331 **	0.317 **
SC	−0.272	−0.136	−0.140	−0.152
GP	0.832	4.251 *	4.041 *	3.371
W × ISW				0.514
W × ST				7.538 **
W × DW				−0.158
W × SD				0.010
W × SO2				0.133
W × IS				−6.200
W × UP				0.013
W × EW				0.689
W × HS				−0.800
W × CS				0.667
W × RD				−0.101
W × SC				1.115 **
W × GP				15,468 ***
ρ			0.054	−0.045
λ		−0.001		
$R^{2}$	0.142	0.115	0.115	0.075
Wald spatial lag				27.69 ***
LR spatial lag				29.16 ***
Wald spatial error				30.60 ***
LR spatial error				30.36 ***
Hausman test				30.99 **

Note: ***, **, * means that the statistics are significant at the levels of 1%, 5%, and 10%, respectively.

**Table 7:** Decomposition results from spatial effects.

Variable	Direct Effect	Indirect Effect	Total Effect
ISW	1.280 ***	0.457	1.737 **
ST	3.298 *	6.960 **	10.259 ***
DW	0.581 ***	−0.174	0.406
SD	−0.289	0.063	−0.226
SO2	−0.149	0.103	−0.046
IS	−16.595 ***	−5.308	−21.903 **
UP	−0.366	0.029	−0.337
EW	0.477	0.640	1.117 ***
HS	1.794 ***	−0.830	0.964
CS	−0.851 *	0.676	−0.175
RD	0.371 **	−0.112	0.205
SC	−0.154	1.078 **	0.924
GP	3.097	14.981 ***	18.079 ***

Note: ***, **, * means that the statistics are significant at the levels of 1%, 5%, and 10%, respectively.

## Data Availability

The data presented in this study are available from the corresponding author.

## Appendix A

**Table A1:** Full name of variables.

**Variable**	**Full Name**
EFT	Environmentally friendly trend
PIT	People’s livelihood improvement trend
ETT	Economic transformation trend
ISW	Comprehensive rate of industrial solid waste utilization
ST	Centralized sewage treatment rate
DW	Harmless disposal rate of domestic waste
SD	Industrial sewage discharge emissions
SO2	Industrial SO2
IS	Industrial soot emissions
UP	Registered unemployed persons in urban areas
EW	Average wage of employed employees
HS	Per capita household deposit savings
CS	Total retail sales of consumer goods per capita
RD	R&D expenditures
SC	Ratio of science and technology expenditure to fiscal expenditure
GP	Green patents
